# Incisional Hernia Depends on More Than Just Mesh Placement

**DOI:** 10.3389/jaws.2024.12954

**Published:** 2024-04-04

**Authors:** M. López-Cano, J. A. Pereira

**Affiliations:** ^1^ Abdominal Wall Surgery Unit, Hospital Universitario Vall d’Hebrón, Universidad Autónoma de Barcelona (UAB), Barcelona, Spain; ^2^ Servicio de Cirugía General, Hospital Universitari del Mar, Departament de Medicina i Ciéncies de la Vida, Universitat Pompeu Fabra, Barcelona, Spain

**Keywords:** hernia, incisional hernia, prevention, treatment, mesh

**FIGURE 1:**
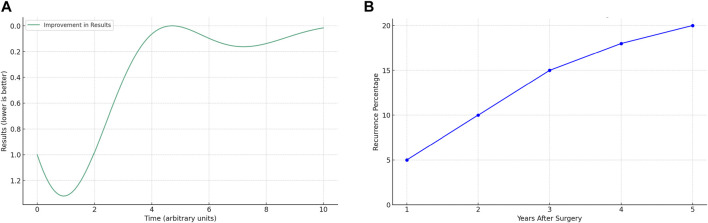
Curves of recurrence evolution: **(A)** Theoretical expected evolution after standardization; **(B)** General evolution observed in the literature data.

Until the end of the 20th century, the repair of incisional hernias (IH) by suture alone was an accepted practice with high recurrence rates reported in observational studies [1–3]. During that period, other observational studies showed lower recurrence rates by adding a permanent synthetic prosthesis to the repair [4–6]. In 2000, a pivotal study was published marking a significant shift in the surgical treatment of IH, being the first randomized analysis comparing the use of a permanent synthetic mesh versus no mesh and its impact on recurrence [7]. The initial results of this research were confirmed over the long term [8], demonstrating a meaningful lower recurrence of IH in patients where a permanent synthetic mesh was added [7, 8]. This initial randomized study strengthened the use of permanent prosthetic mesh as the treatment of choice in the surgical approach to IH. The widespread adoption of mesh in IH treatment likely led to the perception of this entity’s treatment (in terms of reducing recurrence) as a mechanical problem involving only the technical aspects of closing a defect, either with sutures, autologous plasty, or mesh [9]. However, voices soon advocated the view that hernia recurrence should be seen as a much more complex problem, where biology plays a decisive role [10, 11]. Following these arguments [10, 11], if the recurrence of an IH after mesh repair depends only on the technique, what would be the answer to the question: what happens if the process is standardized and excellence in practice is achieved? The answer could be represented by a two-dimensional Cartesian coordinate graph, showing an “S”-shaped curve (Figure 1A). Initially, a high level of variability in the results would be represented by a fluctuating line; over time, these fluctuations would decrease, indicating a more consistent process. Ultimately, the line would stabilize (i.e., plateau), indicating that the best possible results are being consistently achieved over time. However, when analyzing the general information from the literature data, this does not seem to be the case, with a different overall graph being observed (Figure 1B). Thus, in 2003 a population-based analysis (over 10,000 patients) evaluated temporal trends and outcomes after IH repair [12]. The graphs from the study showed a progressive increase in reoperations for IH over time, without a final “plateau” (i.e., recurrence stabilization). Interestingly, the progressive increase was for both patients operated on with and without mesh, with those receiving mesh having a later reintervention. These findings have been confirmed by more recent epidemiological analyses with data from registries [13]. The described context simply supports the considerations made two decades ago [10, 11]. The etiology of IH is multifactorial, and several factors beyond a mere technical aspect can get involved in the event of a recurrence. Furthermore, the use of mesh in IH repair may represent just a “delaying” strategy in the reappearance of IH. In conclusion, the current use of mesh in IH repair may be just a “palliative” treatment for a complex disorder.

In our view, the previous argument not only affects the treatment of IH but can also be applied to its prevention. Thus, one of the most important studies regarding the prevention of IH with non-absorbable synthetic mesh after elective midline laparotomy shows similar long-term curves (5-year) [14]. Preventive mesh only delays the onset of IH. These same long-term results were previously observed in other similar works [15]. Moreover, when it comes to preventing IH with non-absorbable synthetic mesh after emergency midline laparotomy, the scarce long-term follow-up data also show a similar phenomenon where the mesh only delays the onset of the hernia [16]. Finally, it is interesting to note that other studies concerning the closure of the abdominal wall, where the efficacy of meshes in the treatment or prevention of IH is not evaluated, reveal similar curves. Specifically, a long-term analysis of the results of the application of the closure technique recommended in clinical guidelines (i.e., small bites) after both elective and emergency midline laparotomy [17, 18].

In summary, the recurrence of an IH after its treatment or prevention with a permanent synthetic mesh seems to represent only a “delaying” strategy in both elective and emergency surgery. Furthermore, there may be evidence that the closure of a midline laparotomy using the best technique currently recommended in clinical guidelines also only represents a “delaying” strategy in the onset of an IH.

A surgical technique of excellence is key in the outcomes of the treatment or prevention of an IH. However, it seems evident that IH is a complex biological problem. More investment is needed in fundamental research to increase the understanding of an IH genesis. Nevertheless, this fundamental research may take decades to be applied to daily practice, and for this reason, we believe that investment should also be made in clinical research with a view to improve current surgical approaches and prosthetic materials, with the aim of enhancing the best “delaying” strategies of the onset of an IH and preventing the deleterious effects that the footprint (i.e., recurrence, chronic pain, chronic infection, etc.) of techniques and materials can leave on our patients.
